# Cold Atmospheric Plasma, a Novel Approach against Bladder Cancer, with Higher Sensitivity for the High-Grade Cell Line

**DOI:** 10.3390/biology10010041

**Published:** 2021-01-09

**Authors:** Edgar Tavares-da-Silva, Eurico Pereira, Ana S. Pires, Ana R. Neves, Catarina Braz-Guilherme, Inês A. Marques, Ana M. Abrantes, Ana C. Gonçalves, Francisco Caramelo, Rafael Silva-Teixeira, Fernando Mendes, Arnaldo Figueiredo, Maria Filomena Botelho

**Affiliations:** 1 University of Coimbra, Coimbra Institute for Clinical and Biomedical Research (iCBR) area of Environment Genetics and Oncobiology (CIMAGO), Faculty of Medicine, 3000-548 Coimbra, Portugal; ajcfigueiredo@gmail.com; 2 University of Coimbra, Center for Innovative Biomedicine and Biotechnology (CIBB), 3000-548 Coimbra, Portugal; pireslourenco@uc.pt (A.S.P.); inesafmarques@gmail.com (I.A.M.); mabrantes@fmed.uc.pt (A.M.A.); acgoncalves@fmed.uc.pt (A.C.G.); fjmendes@estescoimbra.pt (F.M.); mfbotelho@fmed.uc.pt (M.F.B.); 3 Clinical Academic Center of Coimbra (CACC), 3000-548 Coimbra, Portugal; ritamarneves@gmail.com (A.R.N.); catarinaguilherme_7@hotmail.com (C.B.-G.); fcaramelo@fmed.uc.pt (F.C.); rafaelesteixeira@gmail.com (R.S.-T.); 4 Centro Hospitalar e Universitário de Coimbra (CHUC), Department of Urology and Renal Transplantation, 3004-561 Coimbra, Portugal; 5 University of Coimbra, Coimbra Institute for Clinical and Biomedical Research (iCBR) area of Environment Genetics and Oncobiology (CIMAGO), Biophysics Institute of Faculty of Medicine, 3000-548 Coimbra, Portugal; 6 Project Development Office, Department of Mathematics and Computer Science, Eindhoven University of Technology (TU/e), PO Box 513 5600 MB Eindhoven, The Netherlands; 7 University of Porto, Faculty of Medicine, 4200-319 Porto, Portugal; 8 University of Coimbra, Faculty of Pharmacy, 3000-548 Coimbra, Portugal; 9 University of Coimbra, Coimbra Institute for Clinical and Biomedical Research (iCBR) area of Environment Genetics and Oncobiology (CIMAGO), Laboratory of Oncobiology and Hematology and University Clinic of Hematology of Faculty of Medicine, 3000-548 Coimbra, Portugal; 10 University of Coimbra, Coimbra Institute for Clinical and Biomedical Research (iCBR) area of Environment Genetics and Oncobiology (CIMAGO), Laboratory of Biostatistics and Medical Informatics of Faculty of Medicine, 3000-548 Coimbra, Portugal; 11 Politécnico de Coimbra, ESTeSC, DCBL, Rua 5 de Outubro-SM Bispo, Apartado 7006, 3046-854 Coimbra, Portugal

**Keywords:** cold atmospheric plasma, plasma medicine, bladder cancer, reactive oxygen and nitrogen species, cell death

## Abstract

**Simple Summary:**

Bladder cancer has a high incidence and mortality. Besides this, currently available therapies for this type of cancer have low efficacy and show considerable adverse effects, urging the need of new therapeutic approaches. Cold Atmospheric Plasma treatment presents itself as a promising alternative, having demonstrated antitumor effects against several types of cancer. The present work arises from a multidisciplinary team, namely, medical doctors and researchers, in an attempt to find new therapeutic strategies to fight bladder cancer. Therefore, our main objective is to evaluate Cold Atmospheric Plasma effects against bladder cancer, as well as the mechanisms by which it exerts its effects. The results obtained demonstrate that Cold Atmospheric Plasma treatment has a promising antitumor effect on bladder cancer, with higher sensitivity for the high-grade cell line. This new approach using Cold Atmospheric Plasma for the treatment of bladder cancer presents enormous clinical benefits, since it is able to selectively treat the tumor tissue, sparing the normal urothelium, with an additional glaring positive economic impact, since it entails a decrease in the cost of therapy in comparison with conventional therapeutic options.

**Abstract:**

Antitumor therapies based on Cold Atmospheric Plasma (CAP) are an emerging medical field. In this work, we evaluated CAP effects on bladder cancer. Two bladder cancer cell lines were used, HT-1376 (stage III) and TCCSUP (stage IV). Cell proliferation assays were performed evaluating metabolic activity (MTT assay) and protein content (SRB assay). Cell viability, cell cycle, and mitochondrial membrane potential (Δψ_m_) were assessed using flow cytometry. Reactive oxygen and nitrogen species (RONS) and reduced glutathione (GSH) were evaluated by fluorescence. The assays were carried out with different CAP exposure times. For both cell lines, we obtained a significant reduction in metabolic activity and protein content. There was a decrease in cell viability, as well as a cell cycle arrest in S phase. The Δψ_m_ was significantly reduced. There was an increase in superoxide and nitric oxide and a decrease in peroxide contents, while GSH content did not change. These results were dependent on the exposure time, with small differences for both cell lines, but overall, they were more pronounced in the TCCSUP cell line. CAP showed to have a promising antitumor effect on bladder cancer, with higher sensitivity for the high-grade cell line.

## 1. Introduction

Bladder cancer is the 11th cancer with the highest incidence in the world [1]. About 75% of all bladder tumors are superficial (pTa or pT1) and its treatment is based on endoscopic surgery followed by, in most cases, adjuvant intravesical treatment [2]. This therapeutic combination has undergone little changes in recent decades. In terms of surgery, there are some novelties such as en bloc resections of the tumors [3], that could be an alternative the classic transurethral resections [4] for some patients. However, in terms of intravesical therapy, the therapeutic agents in use are the same since the 1980s, such as mitomycin C and *Bacillus Calmette-Guérin* (BCG) [5,6]. In the European Association of Urology Guidelines for non-muscular invasive bladder cancer [2], like in other guidelines, a single instillation of mitomycin C is indicated postoperatively in most of these patients. After the histology result, patients are classified according to risk (low, intermediate or high). Low-risk patients only receive the postoperative single instillation of mitomycin C. Intermediate risk will take adjuvant therapy with mitomycin C or BCG, according to the therapeutic history and the type of lesions. For high-risk patients, BCG is the only approved adjuvant therapy. Yet, in this group, there is a subgroup of very-high-risk patients that can also be identified, for which the best strategy may be cystectomy. So, intravesical therapeutic agents are of paramount importance in bladder cancer. However, they have significant adverse effects, especially BCG, and progression/relapses are still frequent [7,8,9]. Furthermore, in recent years there have been difficulties in having BCG available. Therefore, there is a need to develop new, safe, and effective therapies for the treatment of bladder cancer.

Plasma is frequently referred to as the fourth state of matter, being a partially ionized gas composed of ions, electrons, and neutral particles [10,11,12]. Quasi-thermal plasmas have been used in medicine for a long time, for example in electrosurgical instruments [13]. Recently, devices capable of producing plasma at room temperature have emerged, the so-called Cold Atmospheric Plasma (CAP). This CAP has been studied for different uses, such as sterilization of materials, wound healing, dental, or neoplasms treatment [14]. By studying different in vitro and in vivo models, it has been shown that CAP is capable of selectively treat neoplasms, sparing normal and healthy tissues [15,16,17]. Indeed, several variables influence CAP selectivity, such as the different types of media used for the culture of different cell lines. To eliminate any bias caused by these experimental variables, more studies addressing this issue must be done. As far as it is known, its main mechanism of action is the production of reactive oxygen and nitrogen species (RONS) [18,19] that leads to an imbalance in the oxidative stress. CAP also induces depolarization of mitochondrial membrane [20], cell cycle arrest [21], and epigenetic alterations [22], among others.

More recently, Plasma Activated Medium (PAM) has been described, a water-based medium that is treated with CAP. PAM acquires characteristics similar to CAP, allowing the selective treatment of neoplasms [23], with two major advantages: (1) PAM can be produced locally in each hospital and can be stored for a few days [24] and (2) its application could easily be held by filling the bladder with PAM.

There are only two studies that demonstrate the effect of CAP on bladder cancer, one in vitro [25] and the other in vivo [26]. However, if the application of CAP or PAM could be able to selectively treat microscopic neoplastic foci, sparing normal urothelium, it is easy to imagine the enormous benefits of its use. Besides that, local production capacity would make it possible to overcome the difficulties that are currently felt in relation to the availability of BCG. Although the desired endpoint is to use PAM as an adjuvant treatment after resection of bladder tumors, as an alternative or adjuctive to mitomycin or BCG, first it is necessary to establish the efficacy of plasma-based therapies in urothelial carcinoma.

In this work, we propose to study the effects of CAP on bladder cancer, particularly in the high-risk/very-high-risk tumors because of high grade tumor (G3) or aggressive variants, for which BCG therapy or even cystectomy are the standard of care. For this, two bladder cancer cell lines were treated with CAP and its anti-tumor capacity were evaluated, as well as the mechanisms by which it exerts its effects, starting from the mechanisms proposed for CAP’s action.

## 2. Materials and Methods

### 2.1. Cell Culture

Human bladder cancer cell lines with different molecular characteristics and belonging to different stages of the disease were used, the HT-1376 cell line (ATCC^®^ CRL-1472™, Manassas, VA, USA) representative of grade III carcinoma and the TCCSUP cell line (ATCC^®^ HTB-5™) representative of grade IV urothelial carcinoma. The cell lines used were obtained from the American Type Culture Collection (ATCC^®^) and maintained in a humidified atmosphere, 37 °C and 5% CO_2_. Both cell lines were cultured in Dulbecco’s Modified Eagle’s Medium, DMEM (Sigma, D5648, Waltham, MA, USA) supplemented with 1% antibiotic (Sigma, A5955), 0.25 mM sodium pyruvate (Gibco, 11360, Waltham, MA, USA), and 10 or 5% FBS (fetal bovine serum) for HT1376 or TCCSUP, respectively.

### 2.2. Cells Treatment

Our group developed a direct current electronic device [27,28,29,30] capable of generating high output voltage through a sterilized needle with 0.9 mm of radius and 40 mm of length (Microlance 3, Becton Dickinson, Franklin Lakes, NJ, USA). When charged, the needle works as an open-air single electrode CAP jet. The equipment was designed to produce an electrical discharge between the tip of the needle and multiwell plates where cell cultures were seeded. An electrically grounded needle was submerged in the culture media. In this arrangement, the cultures act both as target and grounded electrode enabling the plasma generation. The high voltage needle was placed 2 mm above the surface of the cell culture’s medium. The electric current intensity recorded in the needle in a closed circuit was 33 ± 1 µA, the calculated voltage was approximately 4000 ± 121 V and the frequency of pulses was 1000 ± 10 Hz, each pulse with 1.0 ± 0.1 ms of duration. Irradiation of the media did not produce a statistically different alteration in pH media for any exposure time (60 s of CAP treatment pH = 7.41 ± 0.037, 90 s of CAP treatment pH = 7.43 ± 0.061, 120 s of CAP treatment pH = 7.49 ± 0.035, the results are expressed as the mean ± SEM of the pH measurement at 20 °C) nor its temperature, which remained at 26.4 ± 1.043 °C after 120 s of CAP treatment (data not shown).

For the proliferation assays, both cell lines were plated in a 24-well cell culture plate at a concentration of 1 × 10^5^ cell/mL in a volume of 500 µL/well. The cells were allowed to adhere, and then were submitted to CAP treatment, which was generated in open-air, 2 mm above the surface of the cell culture medium, for 15, 30, 60, 90, and 120 s. Upon 24, 48, and 72 h after CAP treatment, metabolic activity was assessed by MTT assay and protein content by SRB assay, to evaluate cell proliferation. 

The viability and cell death profile, the cell cycle, and the mitochondrial membrane potential (Δψ_m_) were studied using flow cytometry 24 h after the CAP treatment. The cells were plated under the same conditions described for the oxidative stress assays and the same CAP exposure times were also used. 

To study the oxidative stress, reactive oxygen and nitrogen species, as well as antioxidant defenses were evaluated. Both cell lines were plated in a 24-well cell culture plate at a concentration of 1 × 10^6^ cell/mL in a volume of 500 µL/well. This cell concentration is not physiological. The cells were allowed to adhere, and then were submitted to CAP treatment 30, 60, and 120 s, times that were selected from the cytotoxicity assays. Upon 2, 6, and 24 h after treatment the intracellular content of hydrogen peroxide, superoxide radical, reduced glutathione, and nitric oxide was evaluated by fluorescence. The hydroxyl radical was also evaluated 24 h after treatment, through the presence of an inhibitor of this reactive specie, D-mannitol. In this assay, the cells were plated under the conditions described for the proliferation assays.

### 2.3. Cell Proliferation Assessment

The metabolic activity was evaluated by the 3-(4,5-dimethylthiazol-2-yl)-2,5-diphenyl tetrazolium bromide (MTT) assay. MTT is a yellow tetrazolium reagent, that is reduced with the formation of purple formazan crystals in the presence of dehydrogenase enzymes located in viable mitochondria [31]. Upon 24, 48, and 72 h after treatment with CAP, MTT protocol was performed as previously described by our group [32]. The absorbance values were determined at 570 and 620 nm wavelengths in a spectrophotometer (EnSpire^®^ Multimode Plate Reader, PerkinElmer^®^, Waltham, MA, USA). Results are presented as the percentage of metabolic activity normalized to the control. 

To evaluate the protein content, it was used the colorimetric sulphorhodamine B (SRB) assay, a negatively charged pink aminoxanthine dye that gives a measure of protein synthesis. The greater the number of cells, the greater amount of dye is taken up, and the greater the absorbance [33]. Upon 24, 48, and 72 h after treatment with CAP, SRB assay was performed according to our published study [34]. Absorbance was quantified at 540 nm with a reference filter of 690 nm in a spectrophotometer (EnSpire^®^ Multimode Plate Reader, PerkinElmer^®^). Results are presented as the percentage of protein content normalized to the control.

### 2.4. Cell Cycle Analysis

To evaluate the effect of CAP on the progression of the cell cycle in both bladder carcinoma cell lines, the labeling with propidium iodide (PI) was used. PI has the ability to interleave in DNA and allows different populations to be obtained in each of the phases of the cell cycle, but it also interleaves with RNA. Thus, in order to obtain a specific DNA labeling, it is necessary to remove the RNA enzymatically using RNase [35]. Cells in the S phase have more DNA than cells in the G_0_/G_1_ phase and cells in the G_2_/M phase have twice the DNA content than cells in the G_0_/G_1_ phase. Sometimes, it is still possible to identify a cell population in pre-G_0_/G_1_ that has less DNA, also called apoptotic peak. Upon 24 h after CAP treatment, cells were detached and 1 × 10^6^ cells were centrifuged for 5 min at 1300× *g*. Cell cycle labeling was performed as previously described [34], using a PI/RNase solution (Immunostep, PI/RNase, Salamanca, Spain). The analysis was performed on a flow cytometer (FACSCalibur, Becton Dickinson), using an excitation wavelength of 488 nm and an emission of 640 nm. Results are presented as the percentage of cells identified in each of the subpopulations: pre-G_0_/G_1_, G_0_/G_1_, S, and G_2_/M.

### 2.5. Viability and Cell Death Profile

To determine the type of cell death induced by CAP in both bladder carcinoma cell lines, the double labeling with annexin V (AV) marked with FITC (fluorescein isothiocyanate) and PI was used. This assay is based on the affinity of AV for phosphatidylserine, which at the beginning of apoptosis is translocated from the inner layer to the outer layer of the plasma membrane, being exposed to the cell surface. PI is used in conjunction with AV since it is intercalated in DNA in cells that have impaired plasma membrane integrity [36]. This double labeling allows to distinguish viable cells (AV^−^/PI^−^) and three different types of cell death: initial apoptosis (AV^+^/PI^−^), late apoptosis/necrosis (AV^+^/PI^+^), and necrosis (AV^−^/PI^+^). Upon 24 h after CAP treatment, cells were detached and 1 × 10^6^ cells were centrifuged for 5 min at 1300× *g*. Then, cells were washed with PBS and incubated with 100 µL of binding buffer (0.01 M Hepes (Sigma, H7523), 0.14 M NaCl and 0.25 mM CaCl2 (Sigma, C4901)), 2.5 µL of AV-FITC (Immunostep, ANXVKF), and 1 µL of PI (Immunostep, ANXVKF) for 15 min at 37 °C, in the dark. After incubation, 400 µL of binding buffer was added and analysis was performed on a flow cytometer (FACSCalibur, Becton Dickinson), using the excitation wavelength of 488 nm and emission wavelengths of 533 nm for the AV-FITC and 640 nm for the PI. Results are presented as the percentage of cells identified in each of the subpopulations: viable cells (V), cells in initial apoptosis (A), cells in late apoptosis/necrosis (A/N), and cells in necrosis (N).

### 2.6. Mitochondrial Membrane Potential Analysis

To evaluate the effect of CAP on the potential of the mitochondrial membrane (Δψ_m_), the fluorescent probe JC-1 (5,5′,6,6′-tetrachloro-1,1′,3,3′-tetraethylbenzimidazolocarbocyanine iodide) was used. JC-1 is a lipophilic and cationic dye with the ability to form aggregates (A) or monomers (M), depending on the state of Δψ_m_. The lower the aggregates/monomers (A/M) ratio, the lower the Δψ_m_, which indicates mitochondrial dysfunction [37,38,39]. Upon 24 h after CAP treatment, cells were detached and after wash with PBS, 1 × 10^6^ cells were labeled with 5 µg/mL of the JC-1 probe (Sigma, T4069) for 15 min at 37 °C, in the dark. Analysis was performed on a flow cytometer (FACSCalibur, Becton Dickinson), using the excitation wavelength of 590 nm for the aggregates and 530 nm for the monomers. Results are presented as the variation in the ratio of the aggregates/monomers (A/M) fluorescence intensities, normalized to the control.

### 2.7. Reactive Oxygen and Nitrogen Species 

The quantification of intracellular peroxides was carried out through the intracellular oxidation of the non-fluorescent probe DCFH_2_-DA (2′,7′-dichlorodihydrofluorescein diacetate), which is internalized in the cells and accumulates preferentially in the cytosol. After deacetylation by intracellular esterases and oxidation in the presence of peroxide, a fluorescent product is formed 2′,7′-dichlorofluorescein (DCF) [40,41]. To estimate the intracellular levels of superoxide radical, the DHE probe (dihydroethidium) was used. This probe easily crosses the cell membrane and is oxidized by the superoxide radical to form a fluorescent red compound that is interleaved in DNA [42]. To determine the intracellular content of nitric oxide, the DAF-FM Diacetate (4-amino-5-methylamino-2′,7′-difluorofluorescein diacetate) probe was used. This probe crosses the cell membrane and is deacetylated by intracellular esterase enzymes, forming the compound DAF-FM. Subsequently, this compound reacts with the nitric oxide molecule, which allows the emission of fluorescence [41].

Upon 2, 6, and 24 h after CAP treatment, 5 × 10^5^ cells were centrifuged for 5 min at 1300× *g*. Then the cells were washed with PBS, centrifuged again for 5 min at 1300× *g* and 1 mL of PBS was added. The cells were incubated with 5 µM of the probe DCFH_2_-DA (Invitrogen, D-399, Carlsbad, CA, USA) for 45 min or with 2 µM of the probe DHE (Sigma, D7008) for 15 min or with 1 μM of the probe DAF-FM Diacetate (Sigma, D2321) for 1 h at 37 °C, in the dark. Finally, the cells were washed with PBS, by centrifugation for 5 min at 1300× *g*. The reading was performed on a spectrophotometer (EnSpire^®^ Multimode Plate Reader, PerkinElmer^®^) with excitation wavelengths of 485 nm and emission of 528 nm for DCFH_2_-DA, 530 nm and emission of 645 nm for DHE and 495 nm and emission of 515 nm for DAF-FM Diacetate. Results are presented as the mean of the fluorescence intensities (MFIs) normalized to the control.

The hydroxyl radical was indirectly evaluated through the use of an inhibitor, D-mannitol, a scavenger for the hydroxyl radical [43,44]. The cells were plated under the same conditions described for the cytotoxicity assays and 2 h before CAP treatment the medium in each well was removed and replaced by fresh medium containing 40 mM of D-mannitol (Sigma, M4125), based on the optimal inhibition concentration described in the literature [45]. In this assay, two controls were used, the negative control consisting of cells where no compound was administered (C), and the mannitol control consisting of cells treated only with mannitol (Cm). Upon 24 h after treatment, cell proliferation was assessed by the MTT assay described previously. Results are presented as the percentage of metabolic activity normalized to the mannitol control (Cm). The percentage data obtained allowed plotting exposure time-response curves.

### 2.8. Antioxidant Defenses 

To determine the intracellular content of reduced glutathione (GSH), a non-enzymatic antioxidant defense, the fluorescent orange mercury compound was used. The probe reacts quickly with GSH, giving rise to a compound that emits fluorescence [46]. Upon 2, 6, and 24 h after CAP treatment 5 × 10^5^ cells were centrifuged for 5 min at 1300× *g*. Then cells were washed with PBS, centrifuged again for 5 min at 1300× *g* and 1 mL of PBS was added. The cells were incubated with 10 μM of orange mercury (Sigma, M7750) for 15 min at 37 °C, in the dark. Finally, the cells were centrifuged for 5 min at 1300× *g* and the reading was performed on a spectrophotometer (EnSpire^®^ Multimode Plate Reader, PerkinElmer^®^) with excitation wavelengths of 485 nm and emission of 590 nm. The results are presented as the MFIs normalized to the control.

### 2.9. Statistical Analysis

Statistical analysis was performed using GraphPad Prism software version 7.00 for Windows (GraphPad Software, La Jolla, CA, USA). The evaluation of the normality of distribution of the quantitative variables was carried out according to the normality test of D’Agostino Pearson. Parametric tests were used in case of a normal distribution and non-parametric tests were used otherwise. Outliers were identified using the ROUT method. The comparison of the different groups of exposure times to CAP was made according to the one-factor Analysis of Variance (ANOVA) test (in the case of normal distribution) or according to the Kruskal Wallis test (otherwise). For multiple comparisons, in case of normal distribution, Dunnett test was used to compare each mean of the exposure time groups with the mean of the control group and Tukey test to compare all the means of all groups with each other, in case of non-normal distribution it was used Dunn’s test. To compare the different times after CAP treatment considering the different exposure times, the two-way ANOVA test was used, using the Tukey test for multiple comparisons. A significance of 0.05 was considered for all comparisons.

## 3. Results

In this study, we investigated the effects of CAP in two bladder cancer cell lines, the HT-1376 (grade III) and the TCCSUP (grade IV). Our focus was to assess cell proliferation, cell viability, and cell death profile, as well as the changes in the cell cycle, the mitochondrial membrane potential, and oxidative stress. In this context, we evaluated the reactive oxygen and nitrogen species involved in the CAP mechanism of action. To make it clearer, from now on, the period of time to which the cells are exposed to treatment with CAP will be called “exposure time,” and the period of time between the end of the exposure to CAP until the evaluation of any parameter at the cellular level will be called “time after CAP treatment.”

### 3.1. CAP Decreases Cell Proliferation

In order to evaluate whether CAP inhibited cell proliferation, the two bladder cancer cell lines were exposed to CAP treatment for 15, 30, 60, 90, and 120 s. MTT and SRB assays were performed 24, 48, and 72 h after CAP treatment.

#### 3.1.1. Cell Metabolic Activity 

**Figure 1:**
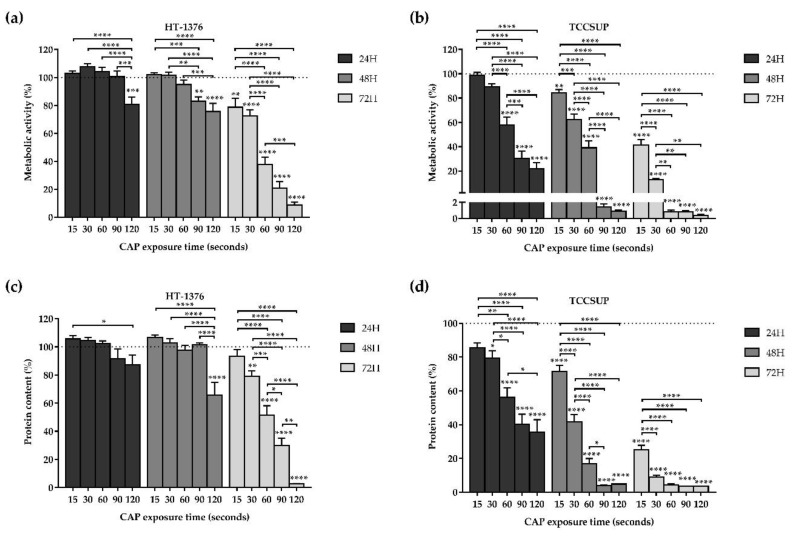
CAP decreases cell metabolic activity and protein content of the HT-1376 (**a**,**c**) and TCCSUP (**b**,**d**) cell lines in an exposure time- and time after CAP treatment-dependent manner. Metabolic activity and protein content were evaluated using MTT and SRB assays, respectively, 24, 48, and 72 h after CAP treatment. Results are expressed as the mean ± SEM of the percentage of metabolic activity or protein content normalized to control (n = 6 independent experiments in triplicate). Statistical differences between exposure times within 24 or 48 or 72 h after the CAP treatment are represented with “*” with horizontal bars and the differences between the exposure times and the control within 24 or 48 or 72 h after the CAP treatment are represented with “*” on top of the respective column, being *p* value reported as * *p* ≤ 0.05, ** *p* ≤ 0.01, *** *p* ≤ 0.001, **** *p* ≤ 0.0001.

In the MTT assay, the metabolic activity decreased in both cell lines depending on the exposure time and time after CAP treatment, as shown in Figure 1a,b. The TCCSUP cell line proved to be more sensitive to CAP treatment when compared to the HT-1376 cell line. 

Comparing the results of the different exposure times with the control, for 24 h after CAP treatment, there was only a significant (*p* = 0.0002) decrease in metabolic activity with a 120 s of exposure time in the HT-1376 cell line (Figure 1a), while in the TCCSUP cell line, there was a significant (*p* < 0.0001) decrease in metabolic activity with an exposure equal to or higher than 60 s (Figure 1b). 

For 48 h after CAP treatment, the longer the exposure time, the lower the metabolic activity with differences statistically significant for exposure times of 90 (*p* = 0.0012) and 120 (*p* < 0.0001) seconds in the case of the HT-1376 cell line (Figure 1a) and exposure times equal to (*p* = 0.0096) or higher than (*p* < 0.0001) 15 s for TCCSUP cell line (Figure 1b). The 90 and 120 s of exposure drastically decreased the TCCSUP metabolic activity to 1.45 ± 0.35% (*p* < 0.0001) and 0.91 ± 0.15% (*p* < 0.0001), respectively. 

Likewise, for 72 h after CAP treatment, in the HT-1376 cell line, an exposure time-dependent response was observed, with differences statistically significant for exposure times equal to (*p* = 0.0040) or higher than (*p* < 0.0001) 15 s of exposure (Figure 1a). With 120 s exposure, the metabolic activity decreased to 8.72 ± 1.99% (*p* < 0.0001). In the TCCSUP cell line, 15 s of exposure was enough to significantly (*p* < 0.0001) decrease the cells metabolic activity (Figure 1b). For exposure times of 60, 90, and 120 s, the metabolic activity decreased drastically to less than 1% (*p* < 0.0001).

Regarding the differences observed between the times after CAP treatment, results show that cell metabolic activity decreases in a time after CAP treatment-dependent manner. For the HT-1376 cell line, only 72 h after CAP treatment with exposure times to CAP equals to or higher than 60 s, it is possible to obtain a decrease in the metabolic activity greater than 50%. Moreover, 72 h after CAP treatment it was observed a significant (*p* < 0.0001) decrease in cell metabolic activity compared with the 24 and 48 h after CAP treatment. The TCCSUP cell line shows to be more sensitive to CAP treatment since, only 24 h after CAP treatment and with an exposure time of 90 s, its metabolic activity is reduced by more than 50%. Upon 48 and 72 h after CAP treatment and with exposure times of 60 (for 72 h after CAP treatment), 90 and 120 s (for 48 and 72 h after CAP treatment), the metabolic activity decreases to values close to 0% (Figure 1b).

#### 3.1.2. Protein Content

Regarding the SRB assay, the protein content decreased in both cell lines depending on the exposure time and time after CAP treatment, as shown in Figure 1c,d. TCCSUP cell line proved to be more sensitive to CAP treatment, corroborating MTT results.

When comparing the exposure times with control, for 24 h after CAP treatment, there was no significant decrease in protein content at any time of exposure in the HT-1376 cell line (Figure 1c). On the other hand, in the TCCSUP cell line, the longer the exposure time, the lower the protein content with differences statistically significant for exposure times equal to (*p* = 0.0195) or higher than (*p* < 0.0001) 30 s of exposure (Figure 1d).

For 48 h after CAP treatment, the protein content of the HT-1376 cell line significantly (*p* < 0.0001) decreased only with an exposure time of 120 s (Figure 1c). Meanwhile, in the TCCSUP cell line, the longer the exposure time, the lower the protein content with differences statistically significant (*p* < 0.0001) for all exposure times (Figure 1d). The 90 and 120 s of exposure time decreased drastically the protein content to 4.04 ± 0.27% (*p* < 0.0001) and 4.81 ± 0.26% (*p* < 0.0001) respectively.

Lastly, for 72 h after CAP treatment, the longer the exposure time, the lower the protein content with differences statistically significant for exposure times equals to (*p* = 0.0077) or higher than (*p* < 0.0001) 30 s of exposure in the HT-1376 cell line (Figure 1c). The 120 s of exposure time reduced the protein content remarkably to 2.56 ± 0.30% (*p* < 0.0001). As for the TCCSUP cell line, all exposure times significantly (*p* < 0.0001) decreased the protein content to less than 50% (Figure 1d).

As demonstrated in the MTT assay, the time after CAP treatment, together with the exposure time, influences the decrease in the cell metabolic activity and also influences the protein content. In the case of the HT-1376 cell line, only 72 h after CAP treatment together with 60, 90, and 120 s of exposure time were capable to decrease the protein content near to or below 50% (Figure 1c). Meanwhile, in the TCCSUP cell line, 24 h after CAP treatment with 90 and 120 s of exposure time were enough to decrease the protein content below 50%, which demonstrates that the TCCSUP cell line is more sensitive to CAP treatment. In addition, with 90 and 120 s of exposure times, 48 and 72 h after CAP treatment, CAP had a similar effect on the protein content, lowering it to values close to 3% (Figure 1d).

Of these assays, the 30, 60, and 120 s of exposure times were selected for the remaining experiments.

### 3.2. CAP Decreases Cell Viability 

The effects of CAP in the viability and cell death profile of the HT-1376 and TCCSUP cell lines were determined, by flow cytometry, 24 h after CAP treatment, using a double labeling with AV and PI. Both cell lines were exposed to CAP treatment for 30, 60, and 120 s. 

**Figure 2:**
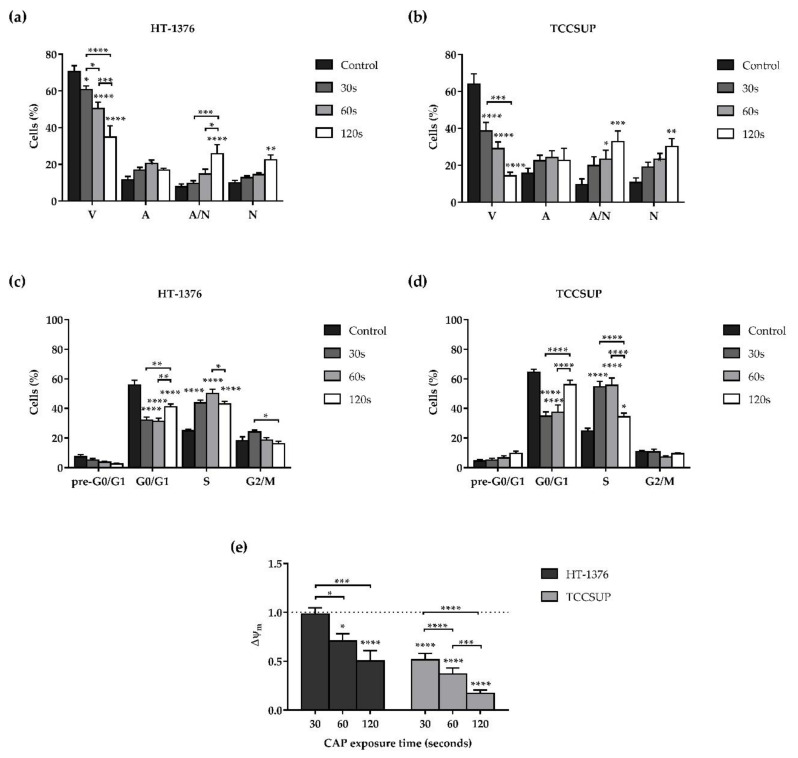
CAP treatment decreases cell viability, induces cell cycle alterations, and depolarization of the Δψ_m_ of HT1376 (**a**,**c**,**e**) and TCCSUP (**b**,**d**,**e**) cell lines in a time of exposure-dependent manner. The viability and cell death profile (n ≥ 4 independent experiments in triplicate), cell cycle progression (n ≥ 3 independent experiments in triplicate), and the Δψ_m_ (n = 4 independent experiments in triplicate) were evaluated by flow cytometry 24 h after CAP treatment. (**a**,**b**) Results of viability and cell death profile are expressed as the mean ± SEM of the percentage of viable cells (V), cells in initial apoptosis (A), cells in late apoptosis/necrosis (A/N), and cells in necrosis (N). (**c**,**d**) Results of cell cycle progression are expressed as the mean ± SEM of the percentage of cells identified in each phase: pre-G_0_/G_1_, G_0_/G_1_, S, and G_2_/M. (**e**) Results of Δψ_m_ are expressed as the mean ± SEM of aggregates/monomers (A/M) ratio normalized to control. *p* value was reported as * *p* ≤ 0.05, ** *p* ≤ 0.01, *** *p* ≤ 0.001, **** *p* ≤ 0.0001. Representative dot plots, from the HT-1376 cell line, of the viability and cell death profile, cell cycle analysis, and the Δψ_m_ assays are presented in Figures S1‒S3 in the Supplementary Materials.

As shown in Figure 2a,b, the longer the exposure time, the lower the cell viability with statistically significant differences for exposure times equal to (*p* = 0.0320) or higher than (*p* < 0.0001) 30 s for HT-1376 cell line (Figure 2a), and for all exposure times (*p* < 0.0001) for TCCSUP cell line (Figure 2b). Once more, the TCCSUP cell line proved to be more sensitive to CAP treatment, since with a 60 s of exposure time the cell viability decreased to 29.07 ± 3.54%, while in the HT-1376 cell line, twice the exposure time (120 s) only decreased the cell viability to 34.93 ± 6.12%. The decrease in cell viability was accompanied by an increase of cells in apoptosis, late apoptosis, and necrosis. In the HT-1376 cell line, the cells in late apoptosis (*p* < 0.0001), as well as the necrotic cells (*p* = 0.0040) significantly increased with 120 s of exposure time (Figure 2a). In the TCCSUP cell line, for exposure times equal to (*p* = 0.0495) or higher than (*p* = 0.0003) 60 s, the percentage of cells in late apoptosis significantly increased, as well as the percentage of cells in necrosis (*p* = 0.0027) for 120 s of exposure time (Figure 2b). In both cell lines, there was no predominant type of cell death for shorter exposure times. However, for longer exposure times, necrosis was the most observed type of cell death.

### 3.3. CAP Induces Alterations on the Cell Cycle

The evaluation of the effect of CAP on the progression of the cell cycle was determined 24 h after CAP treatment, by flow cytometry, using the labeling with PI. Both cell lines were exposed to CAP treatment for 30, 60, and 120 s.

As shown in Figure 2c,d, cell cycle analysis indicates that CAP induced cell cycle arrest of both cell lines in S phase. For the HT-1376 cell line, there was a significant (*p* < 0.0001) increase of cells in the S phase for all exposure times (Figure 2c). Regarding the TCCSUP cell line, there was a significant (*p* < 0.0001) increase of cells in the S phase for exposure times of 30 and 60 s. For 120 s the cells in S phase slightly increase (*p* = 0.0246) compared to control. In addition, in the TCCSUP cell line, after 120 s of CAP treatment, there is a tendency for an increase in the pre-G_0_/G_1_ apoptotic peak (Figure 2d).

### 3.4. CAP Induces Depolarization of the Mitochondrial Membrane Potential 

In order to understand whether CAP induces mitochondrial dysfunction, the effects of CAP in the mitochondrial membrane potential (Δψ_m_) in the two cell lines were determined by flow cytometry, 24 h after CAP treatment, using the probe JC-1. The cells were exposed to CAP treatment for 30, 60, and 120 s. It should be noted that the lower the aggregates/monomers (A/M) ratio, the lower the Δψ_m_, which indicates mitochondrial dysfunction. 

In both cell lines, the longer the exposure time, the lower the Δψ_m_ with differences statistically significant for exposure times of 60 and 120 (*p* < 0.05) seconds in the case of the HT-1376 cell line and for all exposure times (*p* < 0.0001) in the TCCSUP cell line (Figure 2e). The TCCSUP cell line once more proved to be more sensitive to CAP treatment, since 120 s of exposure decreased the Δψ_m_ 2 times in the HT-1376 cell line, while in the TCCSUP cell line a quarter of that exposure time (30 s) also decreased the Δψ_m_ 2 times. Furthermore, 120 s of exposure drastically decreased the Δψ_m_ 5.9 times in the TCCSUP cell line. 

### 3.5. CAP Treatment Induces Alterations in Oxidative Stress of Both Cell Lines 

In order to evaluate the alterations induced by CAP treatment in the oxidative stress by reactive oxygen and nitrogen species involved in the CAP mechanism of action, the two bladder cancer cell lines were exposed to CAP treatment for 30, 60, and 120 s. The intracellular content of peroxides, superoxide radical, as well as GSH, and nitric oxide were evaluated 2, 6, and 24 h after CAP treatment. In addition, the influence of hydroxyl radical was evaluated 24 h after CAP treatment through the presence of an inhibitor of this reactive specie, D-mannitol.

#### 3.5.1. CAP Decreases the Intracellular Content of Peroxides

**Figure 3:**
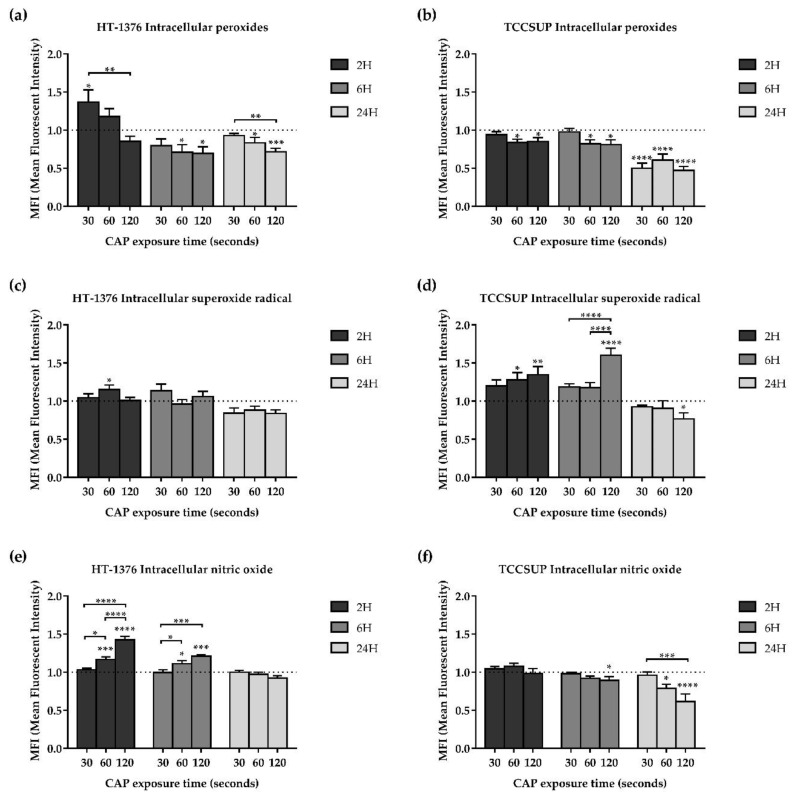
In both HT-1376 and TCCSUP cell lines, the intracellular content of peroxides (**a**,**b**) decreased and the superoxide radical (**c**,**d**) increased, while the nitric oxide increased in the HT-1376 cell line (**e**) and decreased in the TCCSUP cell line (**f**), depending on the exposure time and time after CAP treatment. Intracellular contents of peroxides (n ≥ 4 independent experiments in triplicate), superoxide radical (n ≥ 4 independent experiments in triplicate), and nitric oxide (n ≥ 4 independent experiments in triplicate) were evaluated by fluorescence using the probes DCFH_2_-DA, DHE, and DAF-FM Diacetate, respectively, 2, 6, and 24 h after CAP treatment. Results are expressed as the mean ± SEM of the mean fluorescence intensities (MFIs) normalized to control. Statistical differences between exposure times within 2 or 6 or 24 h after the CAP treatment are represented with “*” with horizontal bars and the differences between the exposure times and the control within 2 or 6 or 24 h after the CAP treatment are represented with “*” on top of the respective column, being *p* value reported as * *p* ≤ 0.05, ** *p* ≤ 0.01, *** *p* ≤ 0.001, **** *p* ≤ 0.0001.

In the HT-1376 cell line, the intracellular content of peroxides significantly (*p* = 0.0320) increased compared to control with 30 s of exposure and 2 h after CAP treatment. However, for longer exposure times and longer times after CAP treatment, intracellular content of peroxides significantly (*p* < 0.05) decreased (Figure 3a). 

Regarding the TCCSUP cell line, for 2 and 6 h after CAP treatment, the intracellular content of peroxides significantly (*p* < 0.05) decreased compared to control with 60 and 120 s of exposure. For 24 h after CAP treatment and with all exposure times, there was a significant (*p* < 0.0001) decrease to approximately half of the intracellular content of peroxides, comparing with control (Figure 3b).

#### 3.5.2. CAP Increases the Intracellular Content of Superoxide Radical

CAP treatment induced an increase of the intracellular content of superoxide radical for short times after CAP treatment in both cell lines, while for longer times after CAP treatment, it induced a decrease in the TCCSUP cell line.

In the HT-1376 cell line, the intracellular content of superoxide radical significantly (*p* = 0.0459) increased compared to control with 60 s of exposure and 2 h after CAP treatment, while no statistical differences were observed in the remaining exposure times and times after CAP treatment (Figure 3c).

Regarding the TCCSUP cell line, for shorter times after CAP treatment (2 and 6 h), the longer the exposure time, the higher the intracellular content of superoxide radical with differences statistically significant for exposure times of 60 (*p* < 0.05) and 120 (*p* < 0.01) seconds relative to control. However, for the longest time after CAP treatment (24 h), superoxide levels significantly decreased (*p* = 0.0480) compared to control with 120 s of exposure. (Figure 3d).

#### 3.5.3. Intracellular Content of Nitric Oxide

CAP treatment induced an increase in the intracellular content of nitric oxide for short times after CAP treatment in the HT-1376 cell line, while, in the TCCSUP cell line the intracellular content of nitric oxide decreased for longer times after CAP treatment.

In the HT-1376 cell line, the intracellular content of nitric oxide significantly (*p* < 0.05) increased compared to control with 60 and 120 s of exposure and with both 2 and 6 h after CAP treatment, while for 24 h after CAP treatment no differences were observed (Figure 3e).

Regarding the TCCSUP cell line, the intracellular content of nitric oxide significantly (*p* < 0.05) decreased compared to control with 60 and 120 s of exposure and with both 6 and 24 h after CAP treatment, while for 2 h after CAP treatment no differences were observed (Figure 3f).

#### 3.5.4. Influence of Hydroxyl Radical

CAP treatment together with a scavenger of the hydroxyl radical decreased the metabolic activity in both cell lines.

**Figure 4:**
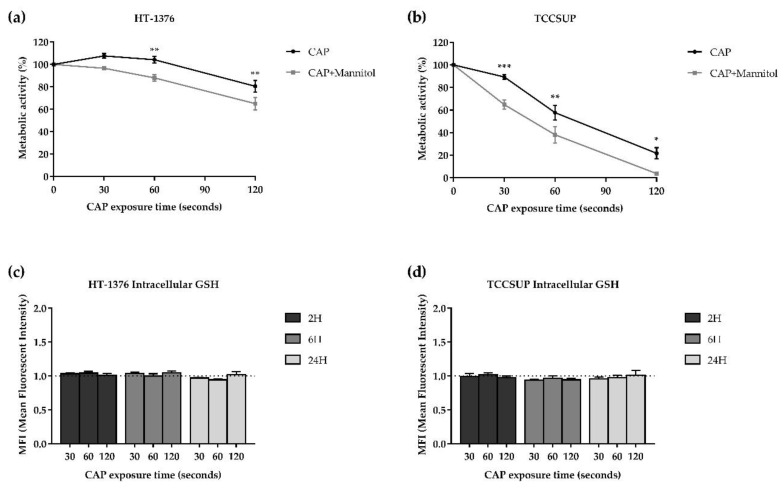
CAP treatment together with D-mannitol decreased the metabolic activity in both HT-1376 (**a**) and TCCSUP (**b**) cell lines. No differences were observed in the intracellular content of GSH in both HT-1376 (**c**) and TCCSUP (**d**) cell lines. The hydroxyl radical was indirectly evaluated using D-mannitol. The cells were treated with CAP or CAP with D-mannitol (CAP + Mannitol), and the cell proliferation was accessed by MTT 24 h after CAP treatment (n = 5 independent experiments in triplicate). Exposure time-response curves were plotted, and the results are expressed as the mean ± SEM of the percentage of metabolic activity normalized to control. Statistical differences between CAP and CAP + Mannitol are represented, being *p* value reported as * *p* ≤ 0.05, ** *p* ≤ 0.01, *** *p* ≤ 0.001. Intracellular content of GSH was evaluated by fluorescence using the fluorescent orange mercury compound, 2, 6, and 24 h after CAP treatment (n ≥ 4 independent experiments in triplicate). Results are expressed as the mean ± SEM of the MFIs normalized to control.

In the HT-1376 cell line, the combined treatment significantly (*p* < 0.01) decreased the metabolic activity with 60 and 120 s of exposure time compared to CAP treatment alone (Figure 4a). The same was true for the TCCSUP cell line (*p* < 0.05), but with 30, 60, and 120 s of exposure time (Figure 4b). It is of note that D-mannitol alone did not interfere with the metabolic activity of the two cell lines (results not shown).

#### 3.5.5. CAP Does Not Affect the Intracellular Content of GSH

As shown in Figure 4c,d, CAP treatment did not induce any alterations in the intracellular content of GSH in both cell lines, since there were no statistically significant differences between control and the CAP exposure times, for any time after CAP treatment.

## 4. Discussion

The high incidence and mortality of bladder cancer, as well as the lack of new treatment options combined with the deficiency in efficacy and safety of the existing ones, has forced the development of new, more effective, and safe approaches for the treatment of this cancer [1,47].

Several studies demonstrated that CAP has the ability of inducing antitumor effects on a variety of cancer types, such as hepatoma [48], lymphoma [49], prostate cancer [50], gastric cancer [51], breast cancer [52], brain cancer [53], ovarian cancer [54], skin cancer [55], head and neck cancer [56], colorectal cancer [57], and lung cancer [58]. For bladder cancer, Mohades et al. already demonstrated a decrease in cell viability and induction of apoptosis after CAP treatment but only in one cell line [25], while Keidar et al. demonstrated tumor ablation in a mouse xenograft model of bladder cancer [26]. In these two studies, only the SCaBER cell line was used. This cell line is representative of squamous cell carcinoma, which only accounts for 2%–5% of bladder tumors [59]. The present study demonstrates the promising application of CAP in bladder cancer treatment through several experiments carried out on two bladder cancer cell lines belonging to different stages of the disease.

Considered together the results for MTT and SRB assays, we can conclude that CAP treatment significantly reduces cell proliferation in a time of exposure- as well as a time after CAP treatment-dependent manner. The same was observed by Wang et al. and Guerrero-Preston et al. in breast cancer and head and neck cancer cell lines, respectively [56,60]. We can also see that the effects of CAP are also dependent on the cell line, and in this case, it proved to be more effective on the high-grade cell line (TCCSUP). Therefore, the optimal time of exposure for maximum efficacy must consider the cell line and its grade.

According to the literature, CAP treatment can induce autophagy [50], senescence [21,61], apoptosis, or necrosis [62,63,64,65] in a time of exposure-dependent manner. However, other factors may also be involved in the cell fate after CAP treatment, such as cell type and plasma source [17]. The CAP treatment when applied to bladder cancer cell lines demonstrated an induction of apoptosis and necrosis. For shorter exposure times, there was no predominant type of cell death, while, for longer exposure times, there was an increase of necrosis. What could be explained by the oxidative stress impairment is that a more intense oxidative stimulus is associated with necrosis.

After evaluating the alterations on the cell cycle, CAP treatment induced cell cycle arrest in the S phase in both cell lines. Vandamme et al. as already demonstrated cell cycle arrest in S and G_2_/M phases [66]. Volotskova et al. observed alterations in the S phase, but this study and other authors have reported cell cycle arrest mostly in the G_2_/M phase induced by CAP treatment [67,68]. Interestingly, the cell proliferation decrease previously observed after MTT and SRB assays should be translated in a cell cycle blockade in G_0_/G_1_ phase, which was not observed. According to the Catalogue of Somatic Mutations in Cancer (COSMIC) (https://cancer.sanger.ac.uk/cell_lines) both cell lines used express *RB1* and *TP53* mutations. RB1 and P53 proteins are involved in major signaling pathways that regulate G_1_ phase, influencing the decision on whether or not a cell enters in S phase [69]. However, the presence of mutations in genes encoding key cell cycle proteins, like RB1 and P53 can lead to aberrant activation of CDKs and to failures in activating cell cycle checkpoints [70]. The cell cycle arrest in S phase could be explained by the ability of CAP to induce DNA damage, including DNA double-strand breaks that will cause DNA fragmentation preventing the cells from passing the DNA replication phase and/or making this phase slower [17]. TCCSUP cells demonstrated a completely different profile in the 120 s of exposure, this could be due to 85% of the cells were dead and only 15% were viable and reflected this cell cycle profile. Here it is possible to see an increase in the apoptotic peak that may indicate cell death by apoptosis.

As previously reported, CAP treatment induces depolarization of the mitochondrial membrane and thus mitochondria-mediated cell death [20,71]. In our results, we also observed depolarization of the mitochondrial membrane in both cell lines, being the TCCSUP cells the ones that suffered the greatest decrease in the mitochondrial membrane potential, corroborating the results that demonstrated a higher cell death induction in this cell line. These results demonstrate one of the pathways by which CAP induces cell death.

CAP has the ability to selectively induce cell death in cancer cells, in detriment of the normal ones [15,16,17,72,73]. This selectivity is mainly due to the imbalance in the oxidative stress caused by CAP and to the differences between normal and cancer cells that facilitates RONS penetration into the cell, such as the higher levels of aquaporins in cancer cells, and the membrane lipid structure, which is influenced by the lower levels of cholesterol in cancer cells [16,17,72].

In the present study, this imbalance in the cellular stress caused by CAP was studied in order to understand which RONS plays a key role in the CAP mechanism of action. We observed that the intracellular content of peroxides decreased in both cell lines depending on the exposure time and time after CAP treatment, contrary to what has been reported by other authors, that have seen an increase in the intracellular content of peroxides after CAP treatment [74,75]. This decrease may be justified by the conversion of the peroxides, namely, hydrogen peroxide, in other more reactive species such as hydroxyl radical, or by their possible detoxification by antioxidant defenses [76]. To prove the possible implication of hydroxyl radical in CAP treatment, cells were treated with both CAP and a scavenger of this reactive specie (mannitol). Compared to the CAP treatment alone, the combination of CAP plus the hydroxyl radical scavenger decreased the metabolic activity, which may indicate that this reactive specie is not involved in the CAP mechanism of action. On the contrary, Kaushik et al. reported a significant protective effect of cancer cell lines by mannitol against CAP treatment, demonstrating the influence of the hydroxyl radical [75]. Thus, this indirect measure to assess the influence of the hydroxyl radical is not the most suitable, because inhibiting a reactive specie will influence the behavior and formation of the remaining ones and induce an imbalance in the oxidative stress itself. More studies must be done in the future.

On the other hand, the intracellular content of superoxide radical increased for shorter times after CAP treatment in both cell lines, being more evident in the TCCSUP cells. These results demonstrate that the superoxide radical may be an important player in the CAP mechanism of action. Superoxide radical can be converted into hydrogen peroxide or can react with nitric oxide and form peroxynitrite radical that is a powerful oxidant capable of inducing membrane damage, DNA fragmentation, lipid peroxidation, cytochrome C release, and, as a consequence, apoptosis and necrosis [64,76,77].

Regarding nitric oxide, our results demonstrate an increase in the intracellular content of nitric oxide in the HT-1376 cells for shorter times after CAP treatment, while in the TCCSUP cells there was a decrease for longer times after CAP treatment. On one hand, as mentioned above, nitric oxide can be involved in the formation of peroxynitrite radical, and, on the other hand, it can have antitumor effects itself, but the mechanisms by which nitric oxide exerts its cytotoxic effects are not entirely clear [17]. Xia et al. demonstrated an increase in the intracellular content of nitric oxide between 3 and 12 h after CAP treatment, as well as an increase of iNOS expression in melanoma cell lines [78]. 

After evaluating the intracellular content of GSH, an antioxidant defense, the results demonstrated no differences between control and CAP exposure times, for any time after CAP treatment. In contrast, Ishaq et al. demonstrated, in melanoma cells, a decrease in the intracellular content of GSH depending on the CAP exposure time [74]. These results demonstrate that the tumor cells were unable to activate their antioxidant machinery to combat oxidative stress induced by CAP and that the decrease in intracellular content of peroxides is not entirely due to their detoxification by the antioxidant defense GSH, but perhaps by the conversion of this reactive specie in others. 

Taken into account the complexity involved in the cell oxidative stress, it is important and mandatory to evaluate other reactive species like the peroxynitrite radical mentioned before, and the other antioxidant defenses, in order to understand how the imbalance in the oxidative stress caused by CAP treatment mediates cell death response. In addition to assessing intracellular content, it is of utmost importance to evaluate the presence of RONS in the extracellular environment in order to comprehend which RONS are delivered by CAP to the cells and which ones play a key role in the CAP mechanism of action.

So far, the present study is the first one to study the effects of CAP treatment in two different bladder cancer cell lines and to demonstrate the greater sensitivity of the higher-grade cell line to CAP treatment. However, more detailed studies highlighting the effects of CAP in bladder cancer and normal cells are needed, in order to completely understand the whole picture of CAP treatment against bladder cancer. With good results, in the future, CAP-based therapies might be an alternative or adjunctive to the actual standard of care. Their applications may be held by different ways. For example, PAM could have a place as sole adjuvant treatment, or as a replacement to saline in the surgical irrigation, or even in the post-operative irrigation. On the other hand, CAP could be applied after a cystectomy or a nephroureterectomy, directly on the surgical bed, to treat microscopic positive margins.

## 5. Conclusions

In our work, we demonstrated the effects of CAP on two bladder cancer cell lines, corresponding to two different stages of the disease. We found a decrease in metabolic activity and protein content for both cell lines. Additionally, there was a significant decrease in cell viability and a cell cycle arrest in both cell lines, mainly in the S phase. One of the main mechanisms of action described for CAP is RNOS production. In the present work, we observed an increase in superoxide and nitric oxide, without changes in GSH levels and with a decrease in peroxide values. The conversion of these reactive species to others such as peroxynitrite is very likely. Another CAP known mechanism of action is the decrease in the mitochondrial membrane potential, which occurred significantly in both cell lines. Some of the effects described are time- (both exposure time and time after CAP treatment) dependent.

There is a need to develop new treatments for bladder tumors. CAP-based therapies may be an alternative in the future. This work is one of the firsts to demonstrate possible antitumoral effects of CAP in urothelial neoplasms, being more pronounced in the more advanced disease cell line (TCCSUP). More works are needed to know better the antitumoral mechanisms and to establish the optimal exposure time to each stage of the disease.

## Data Availability

The data presented in this study will be made available upon request to the corresponding authors.

## Supplementary Materials

The following are available online at https://www.mdpi.com/2079-7737/10/1/41/s1, Figure S1: Representative dot plots, from the HT-1376 cell line, of the viability and cell death profile assay, Figure S2: Representative dot plots, from the HT-1376 cell line, of the cell cycle analysis assay, Figure S3: Representative dot plots, from the HT-1376 cell line, of the mitochondrial membrane potential analysis assay.
