# Supplementary material for: Cold Atmospheric Plasma, a Novel Approach against Bladder Cancer, with Higher Sensitivity for the High-Grade Cell Line
Source: Biology (Basel). 2021 Jan 9;10(1):41. doi: 10.3390/biology10010041 (PMC7828061; doi:10.3390/biology10010041)
Supplement: Supplementary file 1 [file biology-10-00041-s001.pdf]

Supplementary Materials

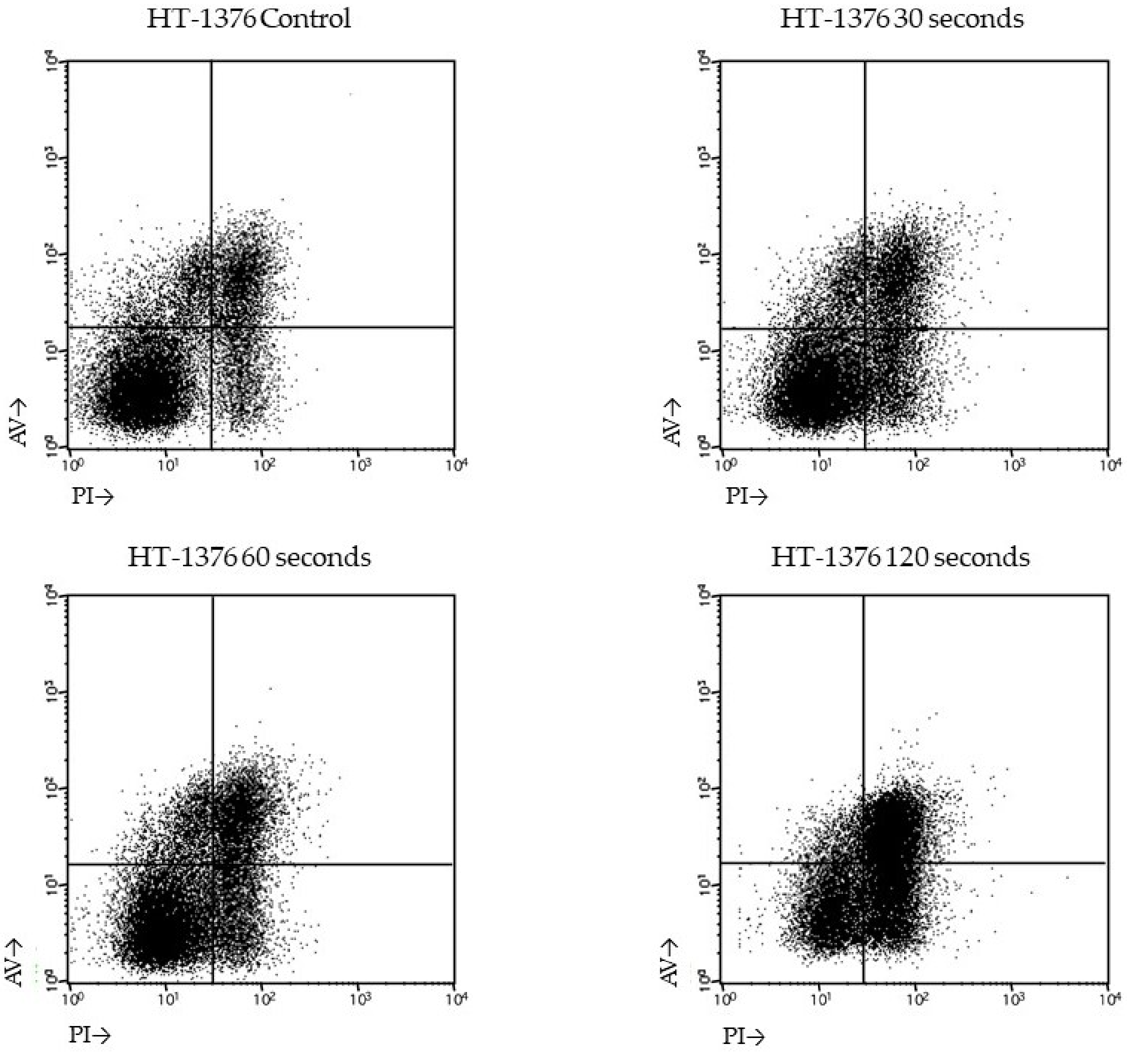

Figure S1. Representative dot plots, from the HT-1376 cell line, of the viability and cell death profile assay. The viability and cell death profile were evaluated by flow cytometry 24 hours after CAP treatment using the double labeling with annexin V (AV) marked with FITC (fluorescein isothiocyanate) and propidium iodide (PI).

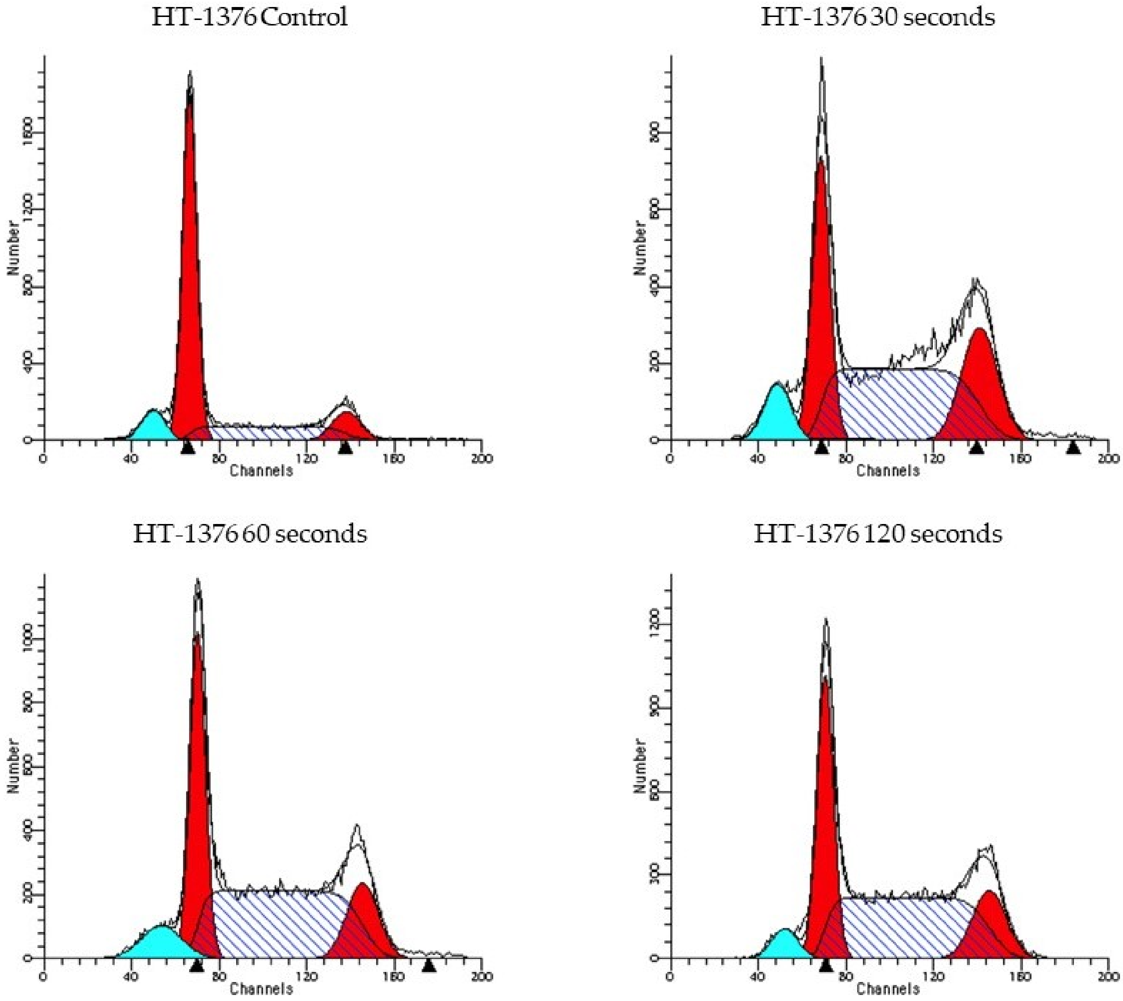

Figure S2. Representative dot plots, from the HT-1376 cell line, of the cell cycle analysis assay. The cell cycle progression was evaluated by flow cytometry 24 hours after CAP treatment using the labeling with PI.

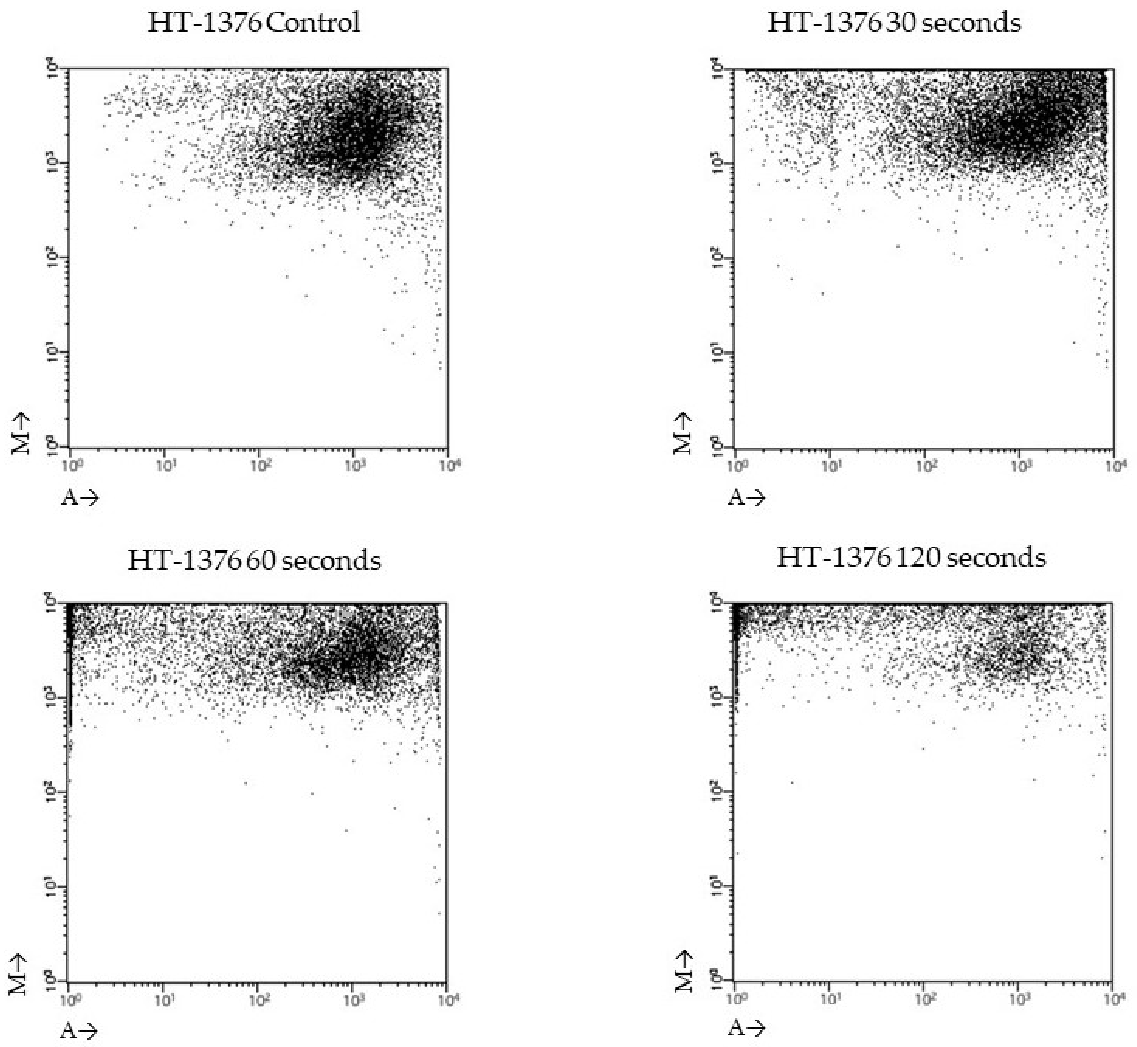

Figure S3. Representative dot plots, from the HT-1376 cell line, of the mitochondrial membrane potential analysis assay. The mitochondrial membrane potential was evaluated by flow cytometry 24 hours after CAP treatment using the fluorescent probe JC-1 (5,5′,6,6′-tetrachloro-1,1′,3,3′-tetraethylbenzimidazolocarbocyanine iodide).
